# Impact of seizures and their prophylaxis with antiepileptic drugs on rehabilitation course of patients with traumatic or hemorrhagic brain injury

**DOI:** 10.3389/fneur.2022.1060008

**Published:** 2022-11-11

**Authors:** Valeria Pingue, Chiara Mele, Stefania Biscuola, Antonio Nardone, Sergio Bagnato, Diego Franciotta

**Affiliations:** ^1^Neurorehabilitation and Spinal Unit, Istituti Clinici Scientifici Maugeri IRCCS, Pavia, Italy; ^2^Department of Clinical-Surgical, Diagnostic and Pediatric Sciences, University of Pavia, Pavia, Italy; ^3^Neurorehabilitation Unit, Istituti Clinici Scientifici Maugeri IRCCS, Montescano, PV, Italy; ^4^Unit of Neurophysiology and Unit for Severe Acquired Brain Injuries, Rehabilitation Department, Giuseppe Giglio Foundation, Cefalù, PA, Italy; ^5^IRCCS Mondino Foundation, Pavia, Italy

**Keywords:** neurorehabilitation, traumatic brain injury, hemorrhagic stroke, antiepileptic drugs, epilepsy

## Abstract

**Objective:**

To determine whether, in patients undergoing rehabilitation after traumatic or hemorrhagic brain injury, seizures and the use of antiepileptic drugs (AEDs) negatively impact on functional outcome, and, in turn, whether prophylactic AED therapy can prevent the development of seizures.

**Design:**

Observational retrospective study.

**Setting:**

Highly specialized inpatient neurorehabilitation clinic.

**Participants:**

Patients with traumatic brain injury (TBI), or hemorrhagic stroke (HS) consecutively admitted to our neurorehabilitation unit between January 1, 2009, and December 31, 2018.

**Main measures and variables:**

Patients' demographic data, neurological status (Glasgow Coma Scale), and rehabilitation outcome (Functional Independence Measure scale), both assessed on admission and on discharge, associated neurosurgical procedures (craniectomy, or cranioplasty), AED use, early or late seizures occurrence, and death during hospitalization.

**Results:**

Of 740 patients, 162 (21.9%) had seizures, and prophylactic AEDs were started in 192 (25.9%). Multivariate logistic regression identified severity of brain injury as a risk factor for acute symptomatic seizures (ASS) in HS (OR = 1.800, 95%CI = 1.133–1.859, *p* = 0.013), and for unprovoked seizures (US) in TBI (OR = 1.679, 95%CI = 1.062–2.655, *p* = 0.027). Prophylaxis with AEDs reduced ASS frequency, but, if protracted for months, was associated with US occurrence (HS, *p* < 0.0001; TBI, *p* = 0.0002; vs. untreated patients). Presence of US (β = −0.12; *p* < 0.0001) and prophylaxis with AEDs (β = −0.09; *p* = 0.002), were associated with poor functional outcome, regardless of age, severity of brain insult, and HS vs. TBI subtype.

**Conclusions:**

Severity of brain injury and occurrence of seizures during neurorehabilitation are the main driver of poor outcome in both HS and TBI. The possible detrimental role on the epileptogenic and functional outcome played by seizures prophylaxis with AEDs, nonetheless useful to prevent ASS if administered over the first week after the brain injury, warrants further investigation.

## Introduction

Seizures represent a well-known complication of acute brain injury (ABI). They can occur from the first days up to several years after ABI, with different pathophysiological mechanisms mainly depending on timing (1). In particular, acute symptomatic seizures (ASS), which occur within seven days from ABI, are thought to be directly related to acute and possibly reversible neuronal dysfunction, whereas unprovoked seizures (US), which occur after more than seven days, might follow structural changes in neuronal networks (2). US are associated with a persistent predisposition to generate seizures, being therefore part of an epilepsy condition (1). Treatment of US with antiepileptic drugs (AEDs) soon after their first appearance represents the standard care after ABI, regardless of the etiology (3, 4). Beyond this practice, the post-ABI indiscriminate prescription of AEDs as preventive therapy for seizures remains controversial (3, 5–8), even though they can potentially affect neurological outcomes in many conditions, such as TBI (5), ischemic and hemorrhagic stroke (6), and in ABI patients undergoing craniectomy, or craniotomy (8). As a major concern, the use of AEDs for seizure prophylaxis seems to be irrelevant for the neurological outcome and mortality (6, 9–11), and to increase the frequency of side effects, such as cognitive and behavioral complications (12–14). Based on this evidence, the Brain Trauma Foundation Guidelines (10) recommend the use of AEDs for seizure prophylaxis within the first 7 days after TBI, when the overall benefit should outweigh the complications associated with the therapy (10). In contrast, there are no accepted guidelines to support the clinical practice of seizures prophylaxis with AEDs after ischemic, or hemorrhagic strokes (2, 6, 15), although it seems to be common in real life outside the 7-day recommended treatment window (16, 17), and during the rehabilitation periods (11, 18).

In this study, we conducted a retrospective analysis on the occurrence of seizures and use of AEDs in a large cohort of patients with ABI admitted to rehabilitation, and evaluated the data available from admission to up to 6 months after TBI, or hemorrhagic stroke (HS). The aim was to evaluate the effects of newly occurring seizures and of AED therapy on functional outcome, and the efficacy of AEDs as preventive treatment for seizures.

## Methods

### Population

In this observational retrospective study, we included patients with TBI and hemorrhagic stroke (HS), consecutively admitted to the Neurorehabilitation Unit of the ICS Maugeri of Pavia, Italy, between January 1, 2009, and December 31, 2018.

Inclusion criteria were: (1) age ≥18 years; (2) diagnosis of TBI or HS; (3) admission to our rehabilitation unit within 1 month from the brain injury to continue clinical care and rehabilitation programs started in the intensive care unit (ICU); (4) at least 6 months of observation before the discharge. Exclusion criteria were: (1) previous brain injury, or any other neurological disease; (2) history of epilepsy and/or concurrent use of AEDs; (3) AED prescription for psychiatric disorders; (4) lack of detailed clinical data about the care during the acute phase.

The study design was in conformity with the ethical guidelines of the Declaration of Helsinki and was approved by the local Ethical Committee (ICS Maugeri, ref. 2214 CE). All participants, or their legal guardians signed a written informed consent.

### Variables, data sources and measurements

The following data were collected from electronic clinical records: age at the time of the brain injury, sex, presence of subarachnoid hemorrhage (SAH), associated neurosurgical procedures (namely, craniectomy, or craniotomy), neurological and functional assessments, occurrence of seizures in either the intensive care or rehabilitation units, use and type of AEDs, death during hospitalization. The Glasgow Coma Scale (GCS) was used to assess the neurological status, and the Functional Independence Measure (FIM) scale was used to evaluate the functional outcome. GCS scores measure severity of neurological impairment (a score of 13-15 indicates “mild”, 9-12 “moderate”, 8 or less “severe” brain injury) (19, 20). FIM scores, focused on patients' independence in activities of daily living, measure the level of disability with 13 motor and 5 cognitive items. The total score range is 18–126: 18 represents complete dependence/total need for assistance, and 126 complete independence (21). GCS and FIM scale were administered at admission (T0), and at discharge (T1) from the rehabilitation unit. ΔGCS and ΔFIM scores, corresponding to T1 minus T0 values, were also calculated.

In accordance with the International League Against Epilepsy (ILAE) criteria (1), seizures were classified as ASS if they occurred within 1–7 days after brain injury, or US if they occurred >7 days after brain injury (1). Any paroxysmal event that occurred during hospitalization, either reported by patients, or described by witnesses, underwent clinical assessment and neurophysiology confirmation in all cases, with appropriate monitoring over time. Prophylactic AEDs prescribed in the acute setting, or in rehabilitation unit, were further subdivided into first- or second-generation drugs (22).

### Statistical analysis

Categorical variables were expressed as absolute number and percentage, and compared using the Chi-square test. Continuous variables were expressed as median and interquartile range (IQR). To identify among the variables the potential risk factors for seizures onset and mortality in patients on primary AEDs prophylaxis, a multivariate logistic regression analysis was used. The multivariate models considered as covariates age, sex (male = 0, female = 1), GCS score (classified as mild = 1, moderate = 2, and severe = 3) at admission (T0), presence/absence of seizures, and primary AED prophylaxis. Odds ratio (OR), 95% confidence interval (95% CI), and related significant values obtained from the regression analysis were reported. Multivariate linear regression analysis was used to evaluate the predictive role of seizures and AED therapy on rehabilitation outcome. The multilinear models included the FIM score at discharge (T1) as dependent variable, and age, sex (male = 0, female = 1), GCS score (classified as mild = 1, moderate = 2, and severe = 3) at T0, type of injury (HS = 0, TBI = 1), presence/absence of seizures, and use and type of AEDs (first or second generation) as independent variables. Coefficients of determination (*R*^2^), β coefficients, and *p*-values obtained from the models were reported. A number of different models were tested to avoid collinearity. The models achieving the highest *R*^2^ were reported. Statistical significance was set at 5%. Statistical analyses were performed using SPSS Statistics 21 (IBM Corporation, Somers, NY, USA).

## Results

### Demographic and clinical characteristics

**Figure 1:**
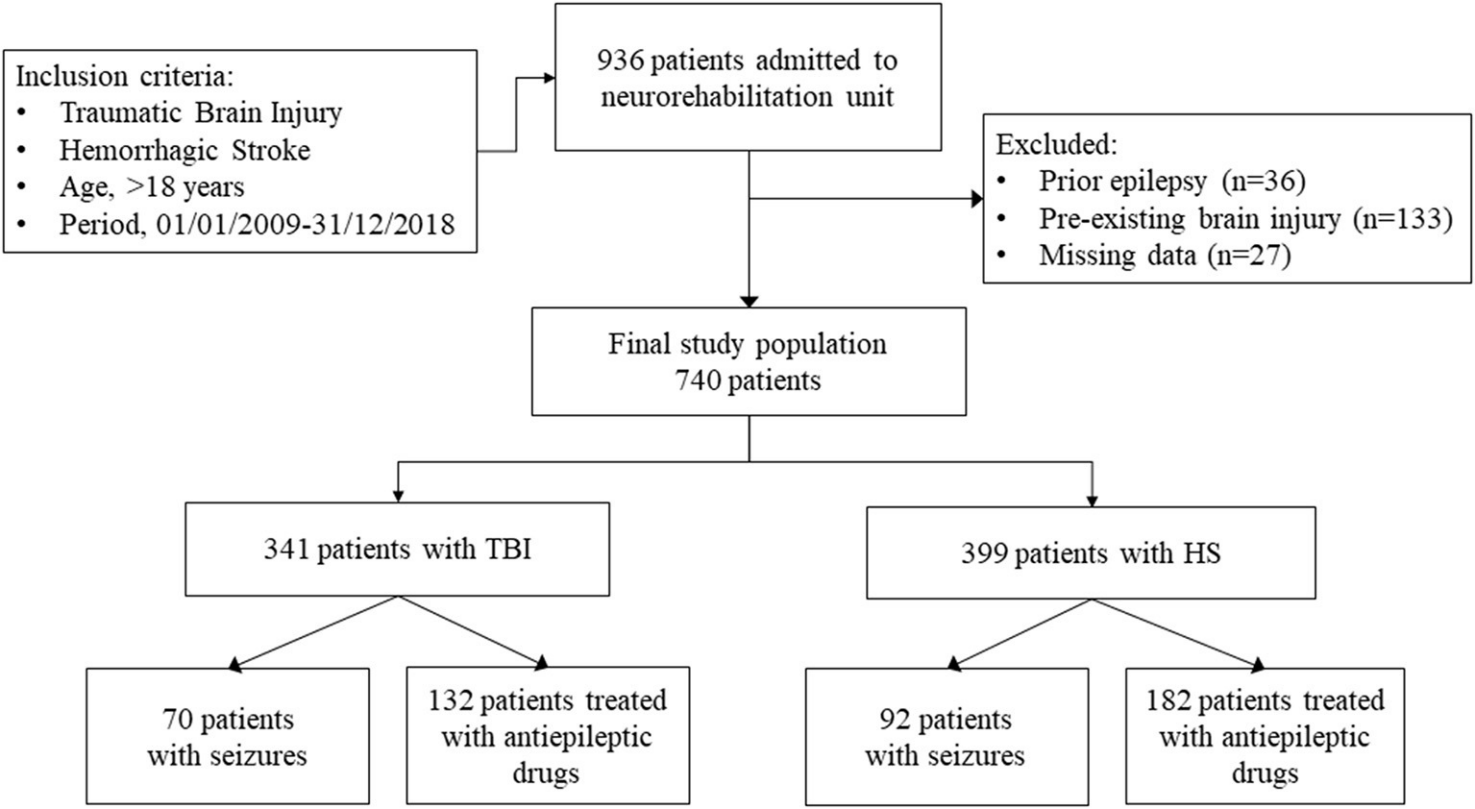
Flow diagram of patients with acquired brain injury enrolled in the study.

We enrolled 740 patients with mild-to-severe ABI due to TBI (46.1%) or HS (53.9%) (Figure 1).

Demographic and clinical characteristics are presented in Table 1.

**Table 1:** Clinical and demographic characteristics of patients with acquired brain injury according to type of injury.

		**All patients (*n =* 740)**	**TBI (*n =* 341; 46.1%)**	**HS (*n =* 399; 53.9%)**	***P-*value**
		***n* (%)**	***n* (%)**	***n* (%)**	
Age (years)	≤65	362 (48.9)	196 (57.5)	166 (41.6)	<0.0001*
	>65	378 (51.1)	145 (42.5)	233 (58.4)	
Sex	Males	465 (62.8)	266 (78.0)	199 (50.0)	<0.0001*
	Females	275 (37.2)	75 (22.0)	200 (50.0)	
GCS score on admission	Mild	256 (34.6)	127 (37.2)	129 (32.9)	0.163
	Moderate	306 (41.4)	136 (39.9)	170 (42.6)	0.455
	Severe	178 (24.0)	78 (22.9)	100 (25.1)	0.491
Subarachnoid hemorrhage		286 (38.6)	135 (39.6)	151 (37.8)	0.649
Craniectomy		196 (26.5)	98 (28.7)	98 (24.6)	0.210
Craniotomy		82 (11.1)	32 (9.4)	50 (12.5)	0.196
Patients with seizures		162 (21.9)	70 (20.5)	92 (23.1)	0.372
Type of seizures	ASS	66 (8.9)	26 (7.6)	40 (10.0)	0.247
	US	78 (10.5)	34 (10)	44 (11.0)	0.718
	ASS+US	18 (2.4)	10 (2.9)	8 (2.0)	0.477
Prophylaxis with antiepileptic drugs		192 (25.9)	82 (24.0)	110 (27.6)	0.312
Antiepileptic drug generation	First	62 (8.4)	26 (7.6)	36 (9.0)	0.509
	Second	235 (31.8)	97 (28.5)	138 (34.6)	0.081
	First and second	17 (2.3)	9 (2.6)	8 (2.0)	0.627
Mortality within 6 months		89 (12.0)	45 (13.2)	44 (11.0)	0.367

Values are expressed as absolute number and percentage; comparisons between groups were performed by χ^2^ test.

* Significant differences.

ASS, acute symptomatic seizures; GCS, Glasgow Coma Scale; HS, hemorrhagic stroke; TBI, traumatic brain injury; US, unprovoked seizures.

Overall, 62.8% of the patients were males (male to female ratio, 1.7:1.0), and 51.1% over 65 years of age. Based on the GCS scores at admission, 34.6% of patients were classified as mild, 41.4% moderate, and 24.0% severe. SAH as a consequence of TBI, or HS was detected in 38.6% of them. De-compressive craniectomy was performed in 26.5% of the patients, and craniotomy in 11.1%. Seizures were focal in most cases and occurred as a likely consequence of ABI in 162 patients (21.9%). ASS were observed in 66 patients (8.9%), US in 78 (10.5%), whereas 18 patients (2.4%) first presented ASS and then US. Overall, primary prophylactic therapy with AEDs was started in 192 patients (25.9%). Second-generation drugs were the most frequently used AEDs for primary prophylaxis (74.4% in TBI, 80% in HS) (Table 2).

**Table 2:** Clinical and demographic characteristics of patients with acquired brain injury according to type of injury and the use of primary prophylaxis with AEDs.

		**TBI** ***n =*** **341**			**HS** ***n =*** **399**		
		**No AEDs** **(*n =* 209; 62.3%)**	**Prophylaxis with AEDs** **(*n =* 82; 24.1%)**	***P-*value**	**No AEDs** **(*n =* 217; 67.9%)**	**Prophylaxis with AEDs** **(*n =* 110; 27.6%)**	***P-*value**
Age (years)	<65	122(58.4)	45(54.9)	0.600	86(39.6)	48(43.6)	0.552
	≥65	87(41.1)	37(45.1)		131(60.7)	62(56.4)	
Sex	Male	162(77.5)	68(82.9)	0.340	115(53.0)	54(49.1)	0.558
	Female	4 (22.5)	14(17.1)		102(40.0)	56(50.9)	
GCS score on admission	Mild	81(38.8)	22(26.8)	0.030*	95(43.8)	18(16.4)	<0.0001*
	Moderate	82(39.2)	30(36.6)	0.504	87(40.1)	49(44.5)	0.476
	Severe	36(17.22)	30(36.6)	0.002	35(16.1)	43(39.1)	<0.0001*
Subarachnoid Hemorrhage		83(39.7)	30(36.6)	0.689	75(34.6)	47(42.1)	0.183
Craniectomy		58(27.7)	45(54.9)	<0.0001*	54 (25.5)	62(56.4)	<0.0001*
Craniotomy		22(10.5)	10(12.2)	0.680	17(7.8)	27(24.5)	<0.0001*
ASS		6(2.9)	2(2.4)	0.999	5(2.3)	1(0.9)	0.668
US		1(2.5)	9(11.0)	<0.0001*	2(0.9)	11(10.0)	0.0002*
ASS +US		0(0.0)	2(2.4)	0.078	0(0.0)	0(0.0)	0.999
AED generation	First	n. a.	13(15.8)	n. a	n. a.	22(20.0)	n. a.
	Second	n. a.	61(74.4)	n. a.	n. a.	88(80.0)	n. a.
Mortality within 6 months		26(12.4)	13(15.8)	0.448	22(10.13)	11(10.0)	0.999

Values are expressed as absolute number and percentage; comparisons between groups were performed by χ^2^ test.

* Significant associations.

AEDs, antiepileptic drugs; ASS, acute symptomatic seizures; GCS, Glasgow Coma Scale; HS, hemorrhagic stroke; n. a., not applicable; TBI, traumatic brain injury; US, unprovoked seizures.

Comparing the two etiologies of brain injury, we found that male sex (*p* < 0.0001) and younger age (*p* < 0.0001) were more frequent in TBI patients. No further differences were found between the two groups in terms of baseline characteristics, neurological assessment with GCS, seizures occurrence, or AED prophylaxis (Table 1).

### Seizures and prophylactic AED therapy

Table 2 compares patients subdivided according to the prescription of the prophylactic antiepileptic therapy. Prophylaxis was more frequently prescribed in patients with severe brain injury (HS, *p* = 002; TBI, *p* < 0.0001), and in those undergoing craniectomy (HS and TBI, *p* < 0.0001). The clinical practice of prophylaxis in patients undergoing craniotomy was limited to those with HS (*p* < 0.0001). Rather unexpectedly, US occurred in patients on primary prevention with AEDs more frequently than in those without AEDs (HS, *p* < 0.0001; TBI, *p* = 0.0002).

Multivariate logistic regression analysis was performed to identify potential risk factors for ASS and US within the 6-month inpatient rehabilitation period (Table 3).

**Table 3:** Potential risk factors for seizures occurrence in patients with acquired brain injury divided by type of injury.

**Covariates**	**ASS**			**US**		
	**OR**	**95% CI**	***P-*value**	**OR**	**95% CI**	***P-*value**
**Hemorrhagic stroke**						
Sex	1.505	0.785–2.887	0.219	1.208	0.663–2.198	0.537
Age >65 years	0.942	0.480–1.848	0.862	0.730	0.399–1.335	0.307
GCS score on admission	1.800	1.133–1.859	0.013*	1.495	0.628–1.495	0.888
ASS	n. a.	n. a.	n. a.	1.112	0.459–2.690	0.814
Prophylactic therapy with AED	0.033	0.004–0.251	0.001*	0.557	0.257–1.206	0.138
Craniectomy	0.550	0.188–1.610	0.276	1.163	0.499–2.713	0.726
Craniotomy	0.543	0.119–2.476	0.431	1.944	0.793–4.766	0.746
**Traumatic brain injury**						
Sex	1.205	0.528–2.751	0.658	0.927	0.408–2.105	0.855
Age >65 years	1.455	0.683–3.097	0.331	0.802	0.389–1.654	0.559
GCS score on admission	0.803	0.481–1.339	0.400	1.679	1.062–2.655	0.027*
ASS	n. a.	n. a.	n. a.	2.735	1.112–6.725	0.028*
Prophylactic therapy with AED	0.322	0.106–0.917	0.044*	0.917	0.410–2.048	0.832
Craniectomy	1.961	0.864–4.451	0.107	3.020	1.412–6.455	0.004*
Craniotomy	2.048	0.680–6.168	0.203	1.011	0.264–3.880	0.987

* Significant associations.

ASS, acute symptomatic seizures; US, unprovoked seizures; GCS, Glasgow Coma Scale; n. a., not applicable; AED, antiepileptic drug.

Severity of the brain injury at admission emerged as a risk factor for ASS in both HS and TBI (HS, *p* = 0.013; TBI, *p* = 0.027). Instead, the presence of ASS emerged as a risk factor for US occurrence after TBI (*p* = 0.028).

No risk factors for US development were identified in patients with HS. In both HS and TBI, the administration of prophylactic therapy with AEDs was associated with lower ASS occurrence (HS, *p* = 0.001; TBI, *p* = 0.045), whereas AEDs as preventive therapy for seizures did not reduce the occurrence of US.

### Functional outcome and mortality

Table 4 shows the data obtained with multilinear regression models for independent predictors of functional outcome.

**Table 4:** Multiple linear regression models for independent predictors of functional outcome according to brain injury, prophylactic use and AED subtype.

**Model 1**	**FIM T1** **(*****R***^**2**^ = **0.483)**	
**Independent variables**	**Beta**	***P-*value**
Sex	0.03	0.226
Age >65 years	−0.22	<0.0001*
GCS score on admission	−0.64	<0.0001*
Etiology of brain injury	−0.02	0.423
Acute symptomatic seizures	−0.04	0.111
Unprovoked seizures	−0.12	<0.0001*
**Model 2**	**FIM T1 (***R*^2^ = **0.484)**	
	**Beta**	***P-*** **value**
Sex	0.03	0.194
Age >65 years	−0.22	<0.0001*
GCS on admission	−0.61	<0.0001*
Etiology of brain injury	−0.02	0.334
Prophylaxis with AEDs	−0.09	0.002*
**Model 3**	**FIM T1 (***R*^2^ = **0.480)**	
	**Beta**	***P-*** **value**
Sex	0.03	0.214
Age >65	−0.22	<0.0001*
GCS on admission	−0.61	<0.0001*
Etiology of brain injury	−0.03	0.328
First generation AEDs	−0.05	0.083
Second generation AEDs	−0.11	<0.0001*

* Significant associations.

AEDs, antiepileptic drugs; GCS, Glasgow coma scale; FIM, functional independence measure.

Development of US, age over 65 years at diagnosis, and worse GCS score (high disability level) at admission independently predicted worse functional outcome at discharge. TBI and ASS occurrence were also predictors of poor functional outcome, as well as the protracted prophylactic therapy with AEDs.

Finally, mortality after ABI at 6 months was documented in 89 patients (12.0%). Multivariate analysis identified older age (*p* < 0.0001), and severity of the brain damage (*p* < 0.0001) as the main predictors of mortality. HS was associated with a lower risk of death during rehabilitation (*p* = 0.035). Instead, no significant association was found between ASS, US, or prophylaxis with AEDs and mortality rate.

## Discussion

In this study we evaluated whether the occurrence of seizures and prophylactic AED therapy could have a negative impact on the 6-month functional outcome in patients with HS, or TBI. We found that the occurrence of US, likely the expression of the brain structural damage with abnormal reorganization of neural networks, and the preventive therapy of seizures with AEDs were predictors of worse functional outcome, independently of age, ABI subtype, and severity of the damage. Notably, this means that the detrimental effect of the prophylaxis with AEDs contributes to worsen the prognosis, in addition to the occurrence of US and severity of the brain injury.

Similar observations on the unfavorable prognostic impact of seizures and epilepsy have been reported in ICH (23), stroke (24), and TBI (5). In particular, ASS could possibly cause additional neurological damage in the early stages of hemorrhagic injury due to the sudden change of blood flow and intracranial pressure (24). Whether US and secondary epilepsy depend on the brain damage severity, which, in turn, drives poor outcome, remains an open question (6). In our cohort, severe neurologic presentations were an independent predictor of ASS in HS, and of US in TBI. Furthermore, ASS occurrence was a risk factor for late seizures in patients with TBI only, as previously reported (4, 5, 10). These patients can benefit from very short courses of antiepileptic medication, limited to within 7 days from the brain injury, to prevent ASS (10), whilst there is no evidence to support seizures prophylaxis after this time frame window, nor after ischemic, or hemorrhagic stroke (6).

Altogether, seizures and brain damage could act synergistically to hamper recovery and drive poor outcome. As a result, severity of the brain insult has prompted the common practice of the prophylactic use of AEDs after ABI. However, this practice is still controversial and discrepancies remain between guideline-driven recommendations and clinical practice (6, 7). In this context, the off-label administration of AEDs is also affected by the lack of indications on therapy duration and the underestimation of adverse effects (6, 25). Our data show that the prophylaxis with AEDs was effective in reducing ASS, but not in reducing the development of US in both the subtypes of ABI. After ABI, ASS are likely the reversible result of a “mechanical” effect that reduces the threshold for seizures (23). In contrast, US depend on complex long-term changes of neural networks and connectivity following brain injury (6, 26). Phenotypic and functional changes in neurons, astro-microglia, and blood-brain barrier likely play a role in these processes (13). In this case, no pharmacological interventions, AEDs included, can prevent the development of epilepsy (6, 9, 13, 27).

Reinforcing the notion of not starting the prevention of seizures and epilepsy with AEDs in ABI, our results also show that this practice is at risk of worse functional outcome, independently of other factors that negatively affect the outcome, such as age, sex, and severity of the brain insult. Besides, the timely AED treatment of newly developing seizures in ABI patients is unlikely to promote their sustained remission (3). In addition to the shown here evidence of detrimental effects on functional outcome, AEDs can also carry cognitive and behavioral side effects, possibly contributing to worsen the outcome and quality of life (13, 14, 28, 29).

Given that first-generation AEDs may potentially affect neural plasticity, thus hindering recovery (9, 30), second-generation AEDs have been increasingly used in ABI (29, 31). These drugs have less side effects and pharmacologic interactions than those of the first-generation AEDs, but there is no clear evidence that they can decrease the percentage of seizure-free patients (32, 33). Second-generation AEDs were preferentially used in our cohort, so that no comparison with the first-generation AEDs in terms of negative impact on functional outcome could be performed. Therefore, our data can only generically support the notion that seizures prophylaxis with AEDs might carry harmful neurotoxicity in ABI.

### Study limitations

This study has some limitations. First, the retrospective design implies the review of clinical files not originally aimed at collecting data for research, with a risk of selection and recall biases, and missing information. For instance, data on location and size of the bleed in HS, useful for a better characterization of the case series, were unavailable. However, the study sample size was very large, and the well-characterized cohort of patients was hospitalized in a tertiary referral center. Second, in this setting, AEDs were prescribed at the clinician's discretion, likely taking into account the severity of the clinical and radiological picture. This could cast doubts on our interpretation of AEDs as a predictor of poor outcomes, being instead a mere indicator of brain damage. But we took this into account, and weighed the multivariate analysis for the severity of the damage. Third, the 6-month follow-up limited the evaluation of functional outcome over the very long-term, but maximum recovery is usually achieved within the first 6 months (34). As for seizures, the chance of their occurrence is very high within this timeframe, since the brain lesions tend to stabilize (35).

## Conclusions

The results of this study suggest that the prophylaxis with AEDs in patients with acquired brain injury should be avoided, due to unfavorable effects on both functional outcome and prevention of epilepsy. Starting the day when the brain injury occurs, their use could be restricted to the first week of hospitalization to prevent ASS. Placebo-controlled double-blind randomized clinical trials are needed to better evaluate efficacy and risks of pharmacological seizure prophylaxis after ABI.

## Data availability statement

The raw data supporting the conclusions of this article will be made available by the authors, without undue reservation.

## Ethics statement

The studies involving human participants were reviewed and approved by ICS Maugeri, Ref. 2214 CE. The patients/participants provided their written informed consent to participate in this study.

## Author contributions

VP: study conception and design, acquisition of data, analysis and interpretation of data, statistical analysis, and drafting the manuscript. CM: statistical analysis and final approval of the version to be published. SB: acquisition of data and drafting manuscript. AN: revision and final approval of the version to be published. SB: critical revision of manuscript for intellectual content and final approval of the version to be published. DF: study design, critical revision of manuscript for intellectual content, and final approval of the version to be published. All authors contributed to the article and approved the submitted version.

## Funding

This work was partially supported by the Italian Ministry of Health under the grant Ricerca Corrente funding schemes to the IRCCS, Istituti Clinici Scientifici Maugeri, and to the IRCCS, Fondazione Mondino, Pavia.

## Conflict of interest

The authors declare that the research was conducted in the absence of any commercial or financial relationships that could be construed as a potential conflict of interest.

## Publisher's note

All claims expressed in this article are solely those of the authors and do not necessarily represent those of their affiliated organizations, or those of the publisher, the editors and the reviewers. Any product that may be evaluated in this article, or claim that may be made by its manufacturer, is not guaranteed or endorsed by the publisher.
